# Sticky Floor and Glass Ceilings in Academic Medicine: Analysis of Race and Gender

**DOI:** 10.7759/cureus.24080

**Published:** 2022-04-12

**Authors:** Chaitanya Shah, Muhammad H Tiwana, Shilpa Chatterjee, Mehr Jain, Ola Lemanowicz, Sabeen Tiwana, Saleh Fares, Javed Siddiqi, Ahmed B Alwazzan, Faisal Khosa

**Affiliations:** 1 Radiology, University of Calgary, Calgary, CAN; 2 Epidemiology and Biostatistics, Western University, London, CAN; 3 Dentistry, Lahore Medical & Dental College, Lahore, PAK; 4 Infectious Diseases, National University Hospital, Queenstown, SGP; 5 Obstetrics and Gynaecology, University of Ottawa, Ottawa, CAN; 6 Medicine, University of British Columbia, Vancouver, CAN; 7 Dentistry, University of British Columbia, Vancouver, CAN; 8 Emergency Medicine, Zayed Military Hospital, Abu Dhabi, ARE; 9 Neurosurgery, Desert Regional Medical Center, Palm Springs, USA; 10 Neurosurgery, Riverside University Health System Medical Center, Moreno Valley, USA; 11 Neurosurgery, Arrowhead Regional Medical Center, Colton, USA; 12 Neurosurgery, California University of Science and Medicine, Colton, USA; 13 Obstetrics and Gynecology, King Abdulaziz University, Jeddah, SAU; 14 Radiology, Vancouver General Hospital, Vancouver, CAN

**Keywords:** race-based differences, gender-based differences, academic rank, academic medicine, retrospective research

## Abstract

Purpose

This paper examines the changes in the representation of women and racial minorities in academic medicine, compares the proportion of minorities in medicine and the general United States (US) population, and discusses potential explanations for observed trends.

Methods

A retrospective cross-sectional analysis of the Association of American Medical Colleges (AAMC) database was done and used to collect data on the gender and race of physicians in academic medicine. Data was collected for instructors, assistant professors, associate professors, full professors, and chairpersons from 2007 to 2018, and trends were presented.

Results

White physicians represented most academic physicians at every academic level, peaking in proportion at 82.74% of chairpersons and were lowest at the level of instructor at 59.30%. A similar distribution existed when gender was compared, with men comprising 84.67% of chairpersons and forming the majority at levels of full, associate, and assistant professors. However, most physicians at the level of instructors are women at 55.44%.

Conclusions

Though women and racial minorities have gained greater representation in academic medicine over the past decade, high-level academic positions are not as accessible to them. Existing efforts of advocacy for women and minority races have proven fruitful over the past decade, but much more work needs to be done.

## Introduction

Gender and racial diversity in medicine improves workforce morale, reduces burnout, enhances patient care, and leads to a higher quality of scientific advancement [1, 2]. Increasing diversity in medical academia also places a greater emphasis on serving populations with poor access to healthcare and increases research on improving healthcare access for marginalized populations [2, 3]. Although recent trend analyses show that more women than men are matriculating in American medical schools, racial minorities are still not well represented amongst medical students. [4, 5]. Additionally, while more women are entering the medical workforce, the same change has been slow to translate into academic medicine, particularly in leadership positions. Lack of progress in equity, diversity, and inclusion has therefore been referred to as an elusive dream and glass ceiling [6].

Given the high value of greater representation in medicine, trend analyses have been conducted for various trainee and physician groups in medicine. These analyses provide insights into the efficacy of representation advocacy and guide the goals of future advocacy efforts. Yu et al. showed that from 1998 to 2007, the greatest representation of women in academic medicine occurred at the lowest levels of the academic ladder, with “instructors” being almost 50% women, whereas only 14.7% of professors were women, with just 9.2% being chairpersons [7]. Racial minorities also follow a similar trend, in that the largest representation of racial minority groups is at the level of instructors or assistant professors at 23.3% and 23.5%, respectively. At the ranks of professors or chairpersons, the representation of racial minorities falls to 10.6% and 9.6%, respectively [7]. Across all specialties of academic medicine, the trend is that women and racial minorities are most prominently represented in the lowest rungs of the academic ladder [7, 8]. These disparities have also been documented across medical schools, multiple specialties, professional societies, and editorial boards of medical journals [9-14].

Since 2007, many advocacy initiatives and social movements in America have been put into action to increase gender and racial representation in medicine, such as the efforts of the black lives matter movement. However, based on our literature review, to date, no study has analyzed trends in gender and racial representation in academic medicine since 2007. This paper examines recent trends in career advancement for women and racial minorities in academic medical from 2007 to 2018 to capture the effects of advocacy efforts in the past decade and a half. This study also aims to provide insights into current diversity gaps in American academics and therefore, guide future advocacy efforts.

## Materials and methods

This was a retrospective data analysis study that was exempt from the Research Ethics Board review. It was based on publicly available data, and the dissemination of results did not identify any individual or generate new forms of identifiable information. The methodology of this study has been validated in recent publications [15, 16]. The data for this study was obtained from the Association of American Medical Colleges (AAMC) website. The AAMC database included demographic data on gender and race for academic positions in US medical school faculties from 2007 to 2018. A data collection tool was developed and tested by a senior author prior to data collection. Data collection was conducted from April 2021 to September 2021. Gender and race data was collected for each of the following academic positions in each medical specialty: instructor, assistant professor, associate professor, professor, and chairperson. Gender variables were defined as “male” and “female”, based on the variables present in the AAMC demographic report categorization of the data. Data was collected for the following racial groups, as defined and categorized by AAMC demographic reports: White, Asian, Black, Hispanic, Multiple Race, unknown, and others. The “others” category includes data from American Indians, Alaskan Natives, Native Hawaiian, or other Pacific Islanders.

Analysis of data

Percentages were used to report trends in gender and race in academic medicine. The percentage of faculty members was averaged over 12 years for each academic position (instructor, assistant professor, associate professor, professor, and chairperson). The absolute change in the number of physicians from 2007 to 2018 was calculated for each gender and racial group.

## Results

Between the years 2007 and 2018, over 46,258 academic medicine positions were added in the United States (US) (Table 1). 

**Table 1:** Absolute change in academic physicians stratified by race and gender from 2007 to 2018 Others include American Indians, Alaskan Natives, Native Hawaiian, Pacific Islanders, etc. "+" denotes increase and "−" denotes decrease

Academic levels	2007, (n)	2018, (n)	Absolute change (n)
All academic physicians:	130,474	176,732	+46,258
Race			
White	92,053	112,894	+20,841
Asian	19,565	34,015	+14,450
Black	4,188	6,288	+2,100
Hispanic	3,978	5,734	+1,756
Multiple race	4,294	7,419	+3,125
Unknown	5,451	8,511	+3,060
Others	945	1,871	+926
Gender			
Male	85,178	103,576	+18,398
Female	44,965	72,917	+27,952
Unreported	331	243	-88

Over the past 12 years, Asian physicians showed the largest increase in the number of chairpersons, associate professors, and instructor positions. At every level of the academic ranks, the most well-represented racial group was Whites (Table 2).

**Table 2:** Race and gender breakdown by academic rank over 12 years (2007-2018) Others include American Indians, Alaskan Natives, Native Hawaiian, Pacific Islanders, etc.

All academic levels	2007, (n)	2008, (n)	2009, (n)	2010, (n)	2011, (n)	2012, (n)	2013, (n)	2014, (n)	2015, (n)	2016, (n)	2017, (n)	2018, (n)	Average over 12 years, (%)
All academic Physicians													
Race													
White	92,053	93,405	95,904	99,183	102,621	104,295	106,847	109,751	112,069	113,242	113,172	112,894	67.19
Asian	19,565	20,638	21,991	23,601	25,339	26,524	27,810	29,555	31,204	32,801	33,911	34,015	17.30
Black	4,188	4,377	4,575	4,782	5,055	5,131	5,385	5,562	5,851	6,022	6,220	6,288	3.37
Hispanic	3,978	4,155	4,309	4,591	4,760	4,917	5,134	5,241	5,378	5,543	5,696	5,734	3.17
Multiple race	4,294	4,505	4,786	5,175	5,597	5,847	6,233	6,591	6,947	7,265	7,448	7,419	3.81
Unknown	5,451	5,899	6,141	6,036	6,145	6,362	6,707	6,790	6,909	7,169	7,579	8,511	4.25
Others	945	1,003	1,079	1,197	1,314	1,410	1,466	1,524	1,665	1,767	1,885	1,871	0.90
Gender													
Male	85,178	86,585	89,050	91,907	95,010	96,369	98,346	100,518	102,424	103,553	103,470	103,576	61.90
Female	44,965	47,026	49,341	52,239	55,407	57,705	60,841	64,130	67,259	69,970	72,238	72,917	37.87
Unreported	331	371	394	419	414	412	395	366	340	286	203	243	0.23
Chairperson													
Race													
White	2,426	2,483	2,508	2,539	2,545	2,579	2,575	2,609	2,625	2,631	2,628	2,574	82.74
Asian	117	134	144	156	172	184	199	208	235	255	266	277	6.25
Black	103	101	98	100	103	103	105	100	105	120	118	126	3.45
Hispanic	86	93	102	110	114	122	126	128	123	117	115	119	3.64
Multiple race	54	56	61	63	64	71	73	77	84	91	90	90	2.34
Unknown	27	26	27	25	26	28	34	35	40	42	49	52	1.10
Others	13	14	13	13	14	16	18	18	17	15	17	15	0.49
Gender													
Male	2,489	2,547	2,555	2,590	2,604	2,650	2,645	2,655	2,674	2,684	2,689	2,656	84.67
Female	337	360	398	416	434	453	485	520	555	587	594	597	15.33
Full professors													
Race													
White	25,893	26,419	27,109	27,621	28,233	28,555	28,972	29,276	29,655	29,620	29,629	29,505	81.15
Asian	2,367	2,514	2,704	2,918	3,140	3,334	3,512	3,736	4,067	4,350	4,631	4,731	9.88
Black	426	437	468	485	510	529	571	602	637	676	703	709	1.59
Hispanic	573	619	656	688	722	766	820	834	872	916	967	972	2.22
Multiple race	805	830	869	911	963	1,010	1,068	1,113	1,174	1,228	1,256	1,275	2.96
Unknown	523	523	545	558	550	568	623	646	681	724	774	825	1.78
Others	129	131	131	136	145	146	146	141	148	153	163	165	0.41
Gender													
Male	25,225	25,628	26,264	26,773	27,295	27,601	27,999	28,272	28,714	28,817	28,809	28,743	78.68
Female	5,406	5,761	6,132	6,452	6,874	7,210	7,624	7,988	8,447	8,795	9,269	9,395	21.10
Unreported	85	84	86	92	94	97	89	88	73	55	45	44	0.23
Associate professors													
Race													
White	20,858	20,976	21,353	21,774	22,205	22,375	22,759	23,057	23,355	23,653	23,797	23,853	71.39
Asian	3,175	3,469	3,772	4,048	4,371	4,744	5,154	5,475	5,827	6,319	6,681	6,733	15.52
Black	734	770	795	825	865	887	903	945	1,019	1,064	1,127	1,160	2.91
Hispanic	788	804	856	901	927	971	1,034	1,073	1,148	1,201	1,222	1,218	3.18
Multiple race	781	844	959	1,060	1,123	1,175	1,267	1,287	1,345	1,410	1,424	1,432	3.68
Unknown	650	723	760	809	829	869	903	938	968	1,000	1,043	1,122	2.78
Others	110	120	133	140	146	153	166	176	205	239	256	262	0.54
Gender													
Male	19,019	19,197	19,612	20,028	20,430	20,666	21,113	21,352	21,779	22,123	22,267	22,319	66.02
Female	8,013	8,435	8,935	9,441	9,954	10,423	10,987	11,528	12,022	12,708	13,241	13,414	33.75
Unreported	64	74	81	88	82	85	86	71	66	55	42	47	0.23
Assistant professors													
Race													
White	34,427	35,353	36,822	38704	40,801	42,185	43,616	45,442	47,063	48,184	48,009	47,948	60.95
Asian	10,456	11,138	11,925	12822	13,724	14,491	15,206	16,254	17,216	18,024	18,421	18,360	21.13
Black	2,361	2,511	2,645	2799	2,971	3,032	3,197	3,279	3,434	3,546	3,619	3,623	4.42
Hispanic	1,992	2,101	2,209	2418	2,541	2,617	2,713	2,726	2,727	2,792	2,822	2,853	3.66
Multiple race	2,211	2,308	2,395	2603	2,813	2,964	3,141	3,379	3,628	3,853	3,985	3,960	4.41
Unknown	2,399	2,634	2,738	2752	2,742	2,875	3,047	3,102	3,183	3,291	3,354	3,974	4.33
Others	487	522	550	599	664	751	801	850	949	1,012	1,080	1,067	1.10
Gender													
Male	32,051	33,073	34,457	36,205	37,990	39,171	40,219	41,713	42,887	43,786	43,474	43,643	56.26
Female	22,121	23,306	24,632	26,284	28,058	29,534	31,305	33,133	35,139	36,767	37,715	38,021	43.48
Unreported	161	188	195	208	208	210	197	186	174	149	101	121	0.26
Instructors													
Race													
White	8,817	8,771	8,740	9,115	9,403	9,271	9,522	9,653	9,500	9,271	9,080	8,995	59.30
Asian	2,796	2,857	2,934	3,106	3,365	3,194	3,171	3,235	3,189	3,162	3,169	3,166	20.09
Black	571	587	588	594	625	591	599	606	634	602	628	652	3.92
Hispanic	525	534	490	486	477	457	447	476	487	490	533	544	3.21
Multiple race	383	416	451	481	571	562	630	674	655	639	628	604	3.58
Unknown	1,112	1,213	1210	1,085	1,102	1,107	1,216	1,262	1,290	1,324	1,424	1,553	8.02
Others	173	200	234	287	328	333	320	321	327	328	340	330	1.88
Gender													
Male	6,909	6,876	6,828	6,991	7,306	6,905	6,987	7,012	6,871	6,640	6,550	6,552	44.43
Female	7,450	7,682	7,792	8,136	8,539	8,593	8,898	9,199	9,190	9,154	9,241	9,268	55.44
Unreported	18	20	27	27	26	17	20	16	21	22	11	24	0.13

Racial trends

**Figure 1:**
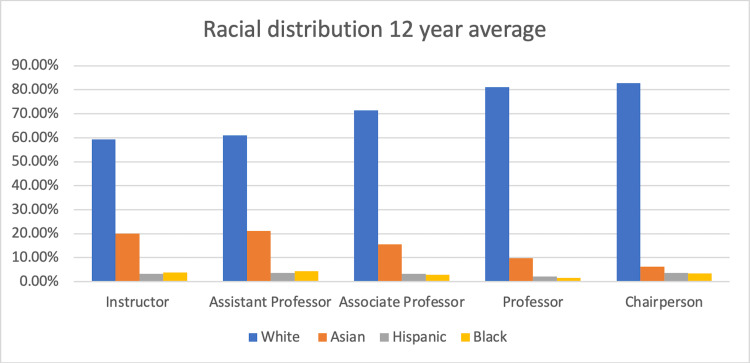
Racial breakdown of academic physicians by position

The proportion of White physicians is lowest at 59.29% in the position of instructor and highest at 82.74% in the position of chairperson. This trend of increasing proportion at higher levels of academia is in sharp contrast to the other racial groups (Figure 1). Asians were in the greatest proportion at the level of instructor and assistant professor at 20.09% and 21.13%, respectively, but taper sharply at the ranks of full professorships and chairpersons at 9.88% and 6.25%, respectively. Hispanics had a relatively flat distribution throughout the different academic positions, going from 3.21% at the position of instructor to a slightly higher 3.64% at the position of chairperson.

Black physicians were most represented at the lowest and highest academic positions, in that the percentage at the level of instructor, assistant professor, and chairperson were highest, while the associate professor and professors were lowest. The percentages in the US general population of White, Asian, Hispanic, and Black are 76.5%, 5.9%, 18.3%, and 13.4%, respectively [17, 18]. Thus, compared to the general US population proportions, Asians are over-represented at every level of the academic ladder, while Hispanic and Black academic physicians are under-represented at every level. For the positions of instructors and assistant professors, White academic physicians are under-represented but are over-represented at the levels of full professors and chairpersons. 

Comparing the number of members in each group at the start and end of the observed period provides an interesting snapshot of the changes that have occurred in academic medicine. Asian physicians have seen the largest relative increase at every academic rank (Table 3), with the largest increases occurring at the highest levels of the academic hierarchy.

**Table 3:** Relative and absolute change in the proportions of each academic rank based on race and sex Others include American Indians, Alaskan Natives, Native Hawaiian, Pacific Islanders, etc. "+" denotes increase, and "−" denotes decrease

All academic levels	2007, (%)	2018, (%)	Relative change (%)	Absolute change (%)
All Academic physicians				
Race				
White	70.55	63.88	-9.45	-6.67
Asian	15.00	19.25	+28.33	+4.25
Black	3.21	3.56	+10.90	+0.35
Hispanic	3.05	3.24	+6.23	+0.19
Multiple race	3.29	4.20	+27.66	+0.91
Unknown	4.18	4.82	+15.31	+0.64
Others	0.72	1.06	+47.22	+0.34
Gender				
Male	65.28	58.61	-10.22	-6.67
Female	34.46	41.26	+19.73	+6.80
Unreported	0.25	0.14	-44.00	-0.11
Chairperson				
Race				
White	85.85	79.13	-7.83	-6.72
Asian	4.14	8.52	+105.80	+4.38
Black	3.64	3.87	+6.32	+0.23
Hispanic	3.04	3.66	+20.39	+0.62
Multiple race	1.91	2.77	+45.03	+0.86
Unknown	0.96	1.60	+66.67	+0.64
Others	0.46	0.46	0.00	0.00
Gender				
Male	88.08	81.65	-7.30	-6.43
Female	11.92	18.35	+53.94	+6.43
Unreported	0.00	0.00	0.00	0.00
Full professors				
Race				
White	84.30	77.27	-8.34	-7.03
Asian	7.71	12.39	+60.70	+4.68
Black	1.39	1.86	+33.81	+0.47
Hispanic	1.87	2.55	+36.36	+0.68
Multiple race	2.62	3.34	+27.48	+0.72
Unknown	1.70	2.16	+27.06	+0.46
Others	0.42	0.43	+2.38	+0.01
Gender				
Male	82.12	75.28	-8.33	-6.84
Female	17.60	24.61	+39.83	+7.01
Unreported	0.28	0.12	-57.14	-0.16
Associate professors				
Race				
White	76.98	66.67	-13.39	-10.31
Asian	11.72	18.82	+60.58	+7.10
Black	2.71	3.24	+19.56	+0.53
Hispanic	2.91	3.40	+16.84	+0.49
Multiple race	2.88	4.00	+38.89	1.12
Unknown	2.40	3.14	+30.83	+0.74
Others	0.41	0.73	+78.05	+0.32
Gender				
Male	70.19	62.38	-11.13	-7.81
Female	29.57	37.49	+26.78	+7.92
Unreported	0.24	0.13	-45.83	-0.11
Assistant professors				
Race				
White	63.36	58.63	-7.47	-4.73
Asian	19.24	22.45	+16.68	+3.21
Black	4.35	4.43	+1.84	+0.08
Hispanic	3.67	3.49	-4.90	-0.18
Multiple race	4.07	4.84	+18.92	+0.77
Unknown	4.42	4.86	+9.95	+0.44
Others	0.90	1.30	+44.44	+0.40
Gender				
Male	58.99	53.36	-9.54	-5.63
Female	40.71	46.49	+14.20	+5.78
Unreported	0.30	0.15	-50.00	-0.15
Instructors				
Race				
White	61.33	56.77	-7.44	-4.56
Asian	19.45	19.98	+2.72	+0.53
Black	3.97	4.12	+3.78	+0.15
Hispanic	3.65	3.43	-6.03	-0.22
Multiple race	2.66	3.81	+43.23	+1.15
Unknown	7.73	9.80	+26.78	+2.07
Others	1.20	2.08	+73.33	+0.88
Gender				
Male	48.06	41.35	-13.96	-6.71
Female	51.82	58.50	+12.89	+6.68
Unreported	0.13	0.15	+15.38	+0.02

From 2007 to 2018, there was a 105% increase in the relative proportion of Asian chairpersons; this was accompanied by an approximately 60% increase in the relative proportion of both Asian full professors and associate professors. This was in stark contrast to the proportion of Hispanic academic physicians with a relative decrease of approximately 5% at the levels of assistant professor and a relative decrease of approximately 6% at the level of instructor. While overall, the relative proportion of Hispanic academic physicians was higher by 6.23%, no other racial minority experienced a decline in numbers at any academic rank. This increase in the proportion of racial minority academic physicians was accompanied by a decrease in the proportion of White academic physicians at every academic rank; however, particularly in the upper echelons of academia, White academic physicians remain over-represented.

Trends in gender

**Figure 2:**
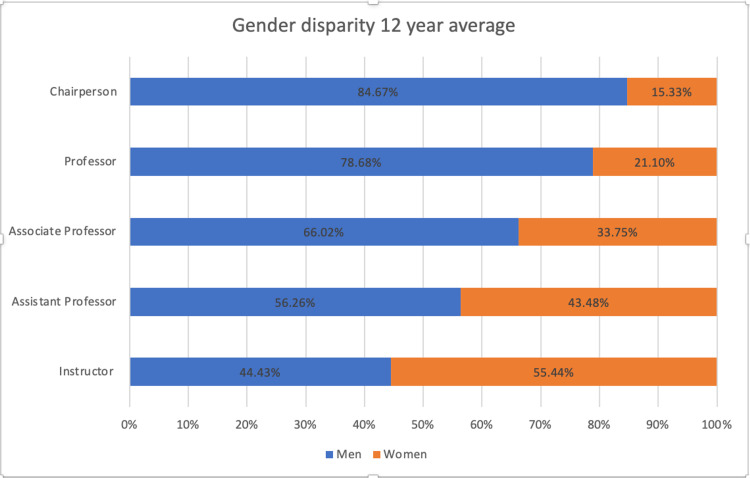
Gender of academic physicians by position

The absolute change from 2007 to 2018 in every academic position was higher in female physicians when compared with male physicians. The proportion of men increased with increasing academic rank (Figure 2).

The only deviation from that trend was at the level of an instructor; over a 12-year average, women represented 55.44% of the group. Beginning at the level of assistant professor, the proportion of men was higher at 56.26%, and this trend continued, peaking at 84.67% at the level of the chairperson. The change in the proportion of 33.75% women at associate professor to 21.10% women at professor showcases an absolute decrease of 12.65% but marks a relative decrease of 62%, the largest drop in proportion for women between academic levels.

The increase of 27,952 women in academic medicine between 2007 and 2018 resulted in a relative increase of 19.73%, while men in academic medicine saw a relative decrease of 10.22%. Women chairpersons, full, associate, and assistant professors, as well as instructors all, saw an increase, with the biggest relative increases being at the levels of chairpersons and full professors. Compared to 2007, in 2018 there was a relative increase of 53.94% in female chairpersons and an increase of 39.83% in female full professors; however, despite the increases, males comprised the majority in higher academic ranks.

## Discussion

The aim of this study was to identify trends in the gender and race of physicians in academic medicine. The most important findings of this study showcased high-level positions in academia have a greater homogeneity in both gender and racial representation, with the highest proportions being represented by males and Whites, respectively. These findings are in keeping with those seen in analysis conducted across all medical specialties, which found that women and racial minorities are generally under-represented in the upper echelons of academic medicine [19, 20]. The one exception to this is Asian academic physicians, who are better represented at all levels of academia compared to other racial minority populations [7, 19]. 

While the reasons for the observed trends are multi-factorial, several compelling arguments have been put forth in the literature which aim to explain the differences seen. While medicine has made great strides in increasing inclusivity among different specialties and at all levels of the academic ladder, these changes have been relatively recent. Thus, more gender and racially diverse cohort of physicians is currently under training and, therefore, potentially decades away from holding positions in the upper rungs of academia such as full professorship or chairperson [21]. However, even at the level of undergraduate medical training, racial diversity can be improved as per a recent study showing the lack of racial diversity over the past four decades in medical students [22]. Secondly, the lack of visible role models for both female and under-represented minority groups has also been proposed as a potential barrier to diversity [23, 24]. Interestingly, while there is an under-representation of females at the level of professors, Kapoor et al. [20] examined factors influencing career advancements such as first author publications or grants from organizations like the National Institutes of Health (NIH) found that women were less likely to have those characteristics and the rates of professorship were not significantly different between men and women after these factors were accounted for. The same study suggested that the under-representation could, therefore, be due to differences in access to research resources or support [20]. Additionally, women reported higher rates of gender discrimination in all specialties except for obstetrics and gynecology, where males reported higher rates of gender discrimination [25]. It showed the predominance of women physicians in the obstetrics and gynecology workforce yet a predominance of White male physicians in tenure positions, senior academic ranks, and leadership positions [25]. These perceptions of discrimination could contribute to certain groups having decreased participation in academic medicine.

To increase diversity in academic medicine and address implicit biases in access to research and leadership positions, current research in the field suggests that blinded reviews be implemented in the research review process. Additionally, improving female representation on editorial boards could be a potential means to prevent bias and improve first authorship among women [26, 27]. In the United Kingdom (UK), the Athena Scientific Women’s Academic Network (SWAN) has approached this issue by recognizing institutions that have focused on improving gender parity in high-level academic positions. This recognition has successfully increased the number of women in leadership positions and the rate at which gender parity is being achieved in academia [28]. Underrepresented racial groups also face barriers to attaining high-level academic positions. Research suggests that promoting mentorship and professional development programs specific to racial and ethnic minorities support more diverse authorship in academic medicine. This leads to more opportunities for members of these populations in the form of promotions and research grants [29]. The success of these interventions could serve as a model for academic institutions as they choose their next steps in improving gender and racial representation in academic medicine. Since succession planning in leadership is vital to continued improvement and lasting excellence in healthcare organizations, a lack of transparency in succession planning is also an important factor that will need redressal [30]

Limitations

This study has its share of limitations. Firstly, only specific genders were included in the study (female and male). Representation of other genders (transgender, non-binary, and others) was not studied. Future studies should assess the representation of all genders as well. There is a limited amount of research on the role that gender and race play in the selection of a career in academic medicine by under-represented groups. This is a relevant area for future research, particularly on the identification of perceived and real barriers to entry and progress in academic medicine, such as access to grant money, resources, and supports needed to publish or the ability to participate in clinical trials. The data did not include indicators of academic productivity such as h-index, number of citations, and the total number of publications. Furthermore, this study did not explore any additive effects of being both a gender and racial minority, which may place them at a further disadvantage.

## Conclusions

Analysis of the AAMC data examining the past 12 years revealed that at higher ranks of academia, there is gender and racial disparity, with a disproportionately high representation of males and Whites. However, between the years 2007 and 2018, under-represented minorities have made large strides at all levels of academia, and the proportions of gender and race in academic medicine are trending towards greater diversity and representation, matching their proportions in the general population. While the causes of the disparities in representation are likely multifactorial, several compelling arguments seek to explain these differences. These results demonstrate a cohort of academic physicians that is increasing in both gender and racial diversity at all levels of academic medicine.
